# Effect of Feminizing Hormone Therapy on QTc Interval: A Secondary Analysis of a Randomized Clinical Trial

**DOI:** 10.1001/jamanetworkopen.2024.3994

**Published:** 2024-03-28

**Authors:** Lachlan M. Angus, Tina Lin, Shalem Y. Leemaqz, Ada S. Cheung

**Affiliations:** 1 Trans Health Research, Department of Medicine, University of Melbourne, Victoria, Australia; 2 Department of Cardiology, Austin Health, Victoria, Australia; 3 College of Medicine and Public Health, Flinders University, South Australia, Australia

## Abstract

This secondary analysis of a randomized clinical trial investigates the effect of spironolactone and cyproterone acetate hormone therapy on the QT interval corrected for heart rate among transgender women and nonbinary or transfeminine individuals.

## Introduction

The adult QT interval has sex-specific reference ranges, reflecting a decrease in the QT interval by 20 ms after male puberty.^1^ The QT interval appears to be modulated by serum testosterone concentrations; it is prolonged in men treated with androgen deprivation therapy for prostate cancer^2^ and shortened in women with hyperandrogenism.^3^ A 2022 cohort study^4^ showed an increased risk of arrhythmia in transgender women compared with cisgender women, but the mechanisms are unclear. We examined the effect of feminizing hormone therapy on the QT interval in transgender women and nonbinary or transfeminine individuals and hypothesized that this would be prolonged due to suppression of serum testosterone levels.

## Methods

In this secondary analysis of a randomized clinical trial, we recruited transgender women and nonbinary or transfeminine individuals commencing feminizing hormone therapy as part of a clinical trial examining antiandrogen medications and feminization in 2020 to 2022 (see trial protocol and statistical analysis plan in Supplement 1; Australian New Zealand Clinical Trials Registry Identifier: 12620000339954). Participants provided written informed consent, and approval was obtained from the Austin Health Human Research Ethics Committee. This study followed the Consolidated Standards of Reporting Trials (CONSORT) reporting guideline.

Participants were treated with standardized estradiol therapy^5^ and randomized to spironolactone 100 mg daily or cyproterone acetate 12.5 mg daily for 6 months. The QT interval was measured once by a single reader (T.L.) from an electrocardiogram and corrected using the Bazett formula at baseline and 6 months. Serum total testosterone and estradiol concentrations were measured in fasting blood samples via liquid chromatography mass spectrometry. Statistical analysis was performed using R statistical software version 4.3.2 (R Project for Statistical Computing) using additional packages (readxl, nlme, emmeans, pbkrtest, and ggplot2). A linear mixed-effects model was used to measure the effect of treatment on the QT interval corrected for heart rate (QTc) over time, adjusting for age and body mass index (calculated as weight in kilograms divided by height in meters squared). Mean difference and corresponding 95% CIs were estimated from predefined post hoc contrasts. *P* < .05 was deemed statistically significant.

## Results

Of 62 individuals enrolled and randomized, 55 women and nonbinary or transfeminine individuals were included in the per protocol analysis (28 and 27 individuals receiving cyproterone acetate and spironolactone, respectively). At 6 months, the overall mean difference in QTc interval was 19.1 ms (95% CI, 13.0-25.3 ms; *P* < .001) (Table), with no between-group difference. No QTc intervals measured were greater than 450 ms, and no arrhythmias were observed. However, 26 participants (51.0%) had an increase in QTc interval greater than 20 ms, and 2 participants (3.9%) had an increase in QTc interval greater than 60 ms, which may be clinically significant (Figure).^6^

**Table.** Participant Characteristics

Characteristic	Participant measure, median (IQR) (N = 55)		*P* value^a^
	Baseline	6 mo	
Age, y	25.3 (21.9-28.7)	NA	NA
Race and ethnicity, No. (%)^b^			
African or Black	2 (3.6)	NA	NA
Asian	4 (7.3)	NA	
White	50 (90.9)	NA	
Other^c^	2 (3.6)	NA	
BMI	26.5 (21.4-30.9)	NA	NA
Serum estradiol, pg/mL	31.8 (25.1-63.9)	104.3 (72.7-158.1)	<.001
Serum total testosterone, ng/dL	478.8 (360.5-643.2)	28.8 (17.3-80.8)	<.001
Serum potassium, mean (SD), mEq/L	4.0 (0.3)	4.0 (0.3)	.97
Manual QTc interval, mean (SD), ms	365.2 (23.2)	384.3 (22.1)	<.001

Abbreviations: BMI, body mass index (calculated as weight in kilograms divided by height in meters squared); NA, not applicable; QTc, QT interval corrected for heart rate.

SI conversion factors: To convert estradiol to picomoles per liter, multiply by 3.671; potassium to millimoles per liter, multiply by 1.0; testosterone to nanomoles per liter, multiply by 0.0347.

^a^ *P* < .05 was deemed statistically significant.

^b^ Participants self-reported race and ethnicity using a survey instrument from the following options and could select multiple options if applicable: Aboriginal and Torres Strait Islander, African/Black, Asian, Hispanic/Latino, Indian subcontinent, Middle Eastern, Pacific Islander, and White. Race and ethnicity were assessed given that participants were expected to be predominantly White, which may limit generalizability of findings.

^c^ Other includes Aboriginal and Torres Strait Islander and Indian subcontinent, which were combined due to low sample size.

**Figure.**
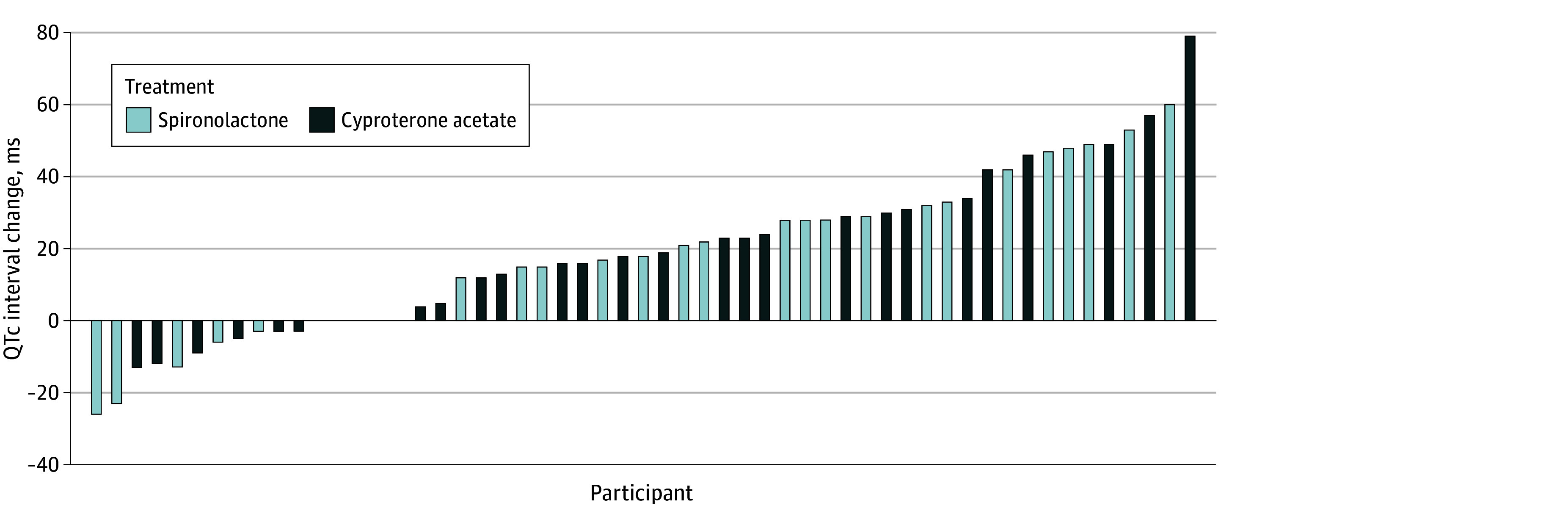
Waterfall Plot of QTc Interval Change Over 6 mo by Participant

The median (IQR) serum estradiol concentration increased from 117.0 (92.0-245.5) pmol/L at baseline to 383.0 (266.9-580.5) pmol/L at 6 months, with no between-group difference .The median (IQR) serum total testosterone concentration decreased from 16.6 (12.5-22.3) nmol/L at baseline to 1.0 (0.6-2.8) nmol/L at 6 months and was lower in the cyproterone acetate than spironolactone group (0.8 [0.6-1.0] vs 1.8 [0.8-4.5] nmol/L; *P* = .001)

## Discussion

In this secondary analysis of a randomized clinical trial of transgender women and nonbinary or transfeminine individuals commencing feminizing hormone therapy, there was statistically significant prolongation of the mean QTc interval by 19.1 ms over 6 months. Despite lower serum total testosterone concentrations in individuals treated with cyproterone acetate (due to potent progestogenic effects), there was no between-group difference in the QTc interval. We speculate that QTc interval may be mediated by tissue-level androgen activity rather than serum testosterone concentration given that cyproterone acetate and spironolactone are peripheral androgen receptor antagonists.

Study limitations include the lack of a control group treated with estradiol monotherapy. Additionally, participants were generally young, White, and free of comorbidity, and the QT interval was measured once by a single reader.

Further research is needed to determine the clinical significance of these findings. Our findings would support use of the female reference range for interpretation of the QTc interval in people taking feminizing hormone therapy in the absence of a larger dataset of transgender women and nonbinary or transfeminine individuals.

## Notes

Supplement 1.
Trial Protocol

Supplement 2.
Data Sharing Statement
