# Supplementary material for: Effect of Feminizing Hormone Therapy on QTc Interval: A Secondary Analysis of a Randomized Clinical Trial
Source: JAMA Netw Open. 2024 Mar 28;7(3):e243994. doi: 10.1001/jamanetworkopen.2024.3994 (PMC10979315; doi:10.1001/jamanetworkopen.2024.3994)
Supplement: Supplement 1. — Trial Protocol [file jamanetwopen-e243994-s001.pdf]

PROTOCOL

A randomised double-blind trial comparing the effectiveness of anti-androgen medications in transgender women

---

Protocol Number: 1.0
Version: 6.0
Date: 11/05/2020

Author/s:
Dr Lachlan Angus, Dr Ada Cheung, Prof Jeffrey Zajac

CONFIDENTIAL

This document is confidential and the property of Austin Health. No part of it may be transmitted, reproduced, published, or used without prior written authorization from the institution.

Statement of Compliance

This document is a protocol for a research project. This study will be conducted in compliance with all stipulation of this protocol, the conditions of the ethics committee approval, the NHMRC National Statement on ethical Conduct in Human Research (2007) and the Note for Guidance on Good Clinical Practice (CPMP/ICH-135/95).

Table of Contents

Contents

Table of Contents 2

1. **Glossary of Abbreviations & Terms** 5

2. **Study Sites** 5

a. Study Location 5

3. **Introduction/Background Information** 6

3.1. Lay Summary 6

3.2. Introduction 6

3.3. Background information 7

4. **Study Objectives** 9

4.1. Hypothesis 9

4.2. Study Aims 9

4.3. Outcome Measures 10

5. **Study Design** 10

5.1. Study Type & Design & Schedule 10

5.2. Standard Care and Additional to Standard Care Procedures 11

5.3. Randomisation 12

5.4. Study methodology 12

6. **Study Population** 14

6.1. Recruitment Procedure 14

6.2. Inclusion Criteria 14

6.3. Exclusion Criteria 14

6.4. Consent 14

7. **Participant Safety and Withdrawal** 14

7.1. Risk Management and Safety 15

7.2. Handling of Withdrawals 15

7.3. Replacements 16

8. Statistical Methods 16

8.1. Sample Size Estimation & Justification 16

8.2. Power Calculations 16

8.3. Statistical Methods To Be Undertaken 16

9. Storage of Blood and Tissue Samples 16

9.1. Details of where samples will be stored, and the type of consent for future use of samples 16

10. Data Security & Handling 17

10.1. Details of where records will be kept & How long will they be stored 17

10.2. Confidentiality and Security 17

10.3. Ancillary data 18

11. References 18

STUDY SYNOPSIS

Title:	A randomised double-blind trial comparing the effectiveness of anti-androgen medications in transgender women
Design:	Double-blind randomised trial
Study Centres:	Austin Health
Hospital:	Austin Health
Study Question:	Is cyproterone acetate more effective than spironolactone in lowering endogenous testosterone levels and increasing breast volume and feminine fat distribution in male-to-female individuals commencing estradiol therapy?
Study Objectives:	1. To compare the effectiveness of cyproterone acetate and spironolactone as an anti-androgen agent in male-to-female transgender individuals commencing estradiol.
Primary Endpoint:	Breast volume
Secondary Endpoint:	1. Percentage of individuals who have total testosterone levels <2 nmol/L (in the female reference range) at 3 and 6 months.<br>2. Fat mass including leg and gynoid regional fat;<br>3. Depression assessment using Patient Health Questionnaire 9;<br>4. Gender Dysphoria using the Gender Preoccupation and Stability Questionnaire.
Inclusion Criteria:	1. Male-female individuals aged 16-80 years<br>2. About to commence treatment with feminising hormone therapy with anti-androgens
Exclusion Criteria:	1. Evidence of androgen deficiency at baseline (T<10nmol/L)<br>2. Any contraindications to spironolactone, cyproterone acetate or E2 therapy
Number of Planned Subjects:	64
Safety considerations:	Regular monitoring for adverse physiological and psychological effects during routine care of cross-sex hormone therapy, including:<br>1. Monitoring of E2 levels<br>2. Monitoring for changes in depression and gender dysphoria

	Ensuring safety when obtaining blood tests.
Statistical Methods:	Generalised linear mixed model with a restricted maximum likelihood function to determine between group differences in the main outcome measures over time.
Subgroups:	Male-to-female intervention group receiving cyproterone acetate; male-to-female intervention group receiving spironolactone.

1. Glossary of Abbreviations & Terms

Abbreviation	Description (using lay language)
CSHT	Cross-sex hormone therapy
DXA	Dual energy X-ray Absorptiometry scan
E2	Estradiol (the main form of estrogen-female hormone)
ECG	Electrocardiograph
GnRH	Gonadotropin-releasing hormone
T	Testosterone (the main male hormone)
LC-MS	Liquid Chromatography Mass Spectrometry
PICF	Participant Information Sheet & Consent Form

2. Study Sites

a. Study Location

Site	Address	Contact Person	Phone	Email
Austin Health	145 Studley Rd, Heidelberg, VIC	Dr Ada Cheung	9496 2260	adac@unimelb.edu.au

3. INTRODUCTION/BACKGROUND INFORMATION

3.1. LAY SUMMARY

Gender identity is at the core of our sense of self, and considerable scientific evidence supports a biological basis. Gender identity is not chosen, but innate, and differs from biological sex which is determined by sex chromosomes and reproductive organs. Transgender people, whose gender identity is markedly and persistently incongruent with their biological sex, almost always experience gender dysphoria. Characterised by severe distress and discomfort, and the feeling of 'having been born in the wrong body', gender dysphoria compels transgender individuals to seek treatment. For many, aligning their physical characteristics with their gender identity through cross-sex hormones becomes an urgent imperative.

Though international clinical guidelines provide protocols for feminising cross-sex hormone therapy, these recommendations are not evidence-based. Anti-androgen medications (commonly known as testosterone blockers) are commonly used with estrogen as feminising cross-sex hormone therapy in transgender women (males-to-females). The goal of feminising hormone therapy is to increase estrogen, and lower testosterone to achieve hormone levels in the female reference range. Estrogen on its own is usually not effective at completely lowering testosterone levels. As such, anti-androgens are commonly used together with estrogen in order to lower or block testosterone, which in turn aids the development of a feminine body shape, breast development, skin and hair changes. The two most commonly used anti-androgen medications in Australia are cyproterone acetate and spironolactone, however no research exists to show which drug is more effective.

The primary aim of this study is to compare the effectiveness of additional anti-androgen treatment (cyproterone acetate versus spironolactone) to cause breast growth, lower testosterone levels and aid development of feminine body composition changes.

Given a profound lack of research, the need for specific, rigorous research to guide hormonal therapy guidelines for the transgender community is very much needed. The results from the study will inform clinical care and treatment choice for both trans and gender diverse individuals undergoing feminising hormonal treatments as well as their treating clinicians.

3.2. INTRODUCTION

Partly due to increasing societal acceptance, there has been a ten-fold increase in transgender individuals seeking cross-sex hormonal therapy in the last 5 years in Victoria, with an estimated 0.6% - 3% of the population identifying as transgender. (1-3) There is a profound

lack of quality research in optimal cross-sex hormone treatment regimens. Our research seeks to address the medical aspects of gender transition: to improve and optimise evidence-based hormonal treatment guidelines, ensuring suitable standards of care for this marginalised population that experiences significant health inequities, with high prevalence of depression and suicidality, difficulty accessing hormone treatment, and issues with treatment side-effects.

Feminising cross-sex hormone therapy with estradiol (E2), improves psychological functioning in male-to-female transgender individuals (4); however, E2 alone is usually insufficient to lower total testosterone (T) levels. As the large majority of individuals (82% in our cohort, similar to international data) do not undergo genital reassignment surgery to remove the testes (responsible for >95% of T production), anti-androgens (cyproterone acetate or spironolactone) are commonly added to E2, lowering or blocking T to aid development of feminine body composition, facial and body hair and skin.

Preliminary data from our retrospective audit (5) of 540 individuals (n=80 male-to-female on E2 for >6 months) suggest that on univariate and multivariable analyses, the cyproterone acetate group had significantly lower total T levels (overall p<0.0002) than the spironolactone group and the E2 alone group. Ninety per-cent of the cyproterone acetate group and 40% of the spironolactone group had total T levels < 2 nmol/L. This preliminary study also found that serum urea level was higher (5.2 vs 4.4 mmol/L, p=0.035) in the spironolactone group. No differences were observed in total daily oral E2 valerate dose, E2 level achieved, blood pressure, creatinine, or liver function. Supporting our findings, a recently published US retrospective audit of 98 male-to-females found only a quarter of those on spironolactone achieved T in the female reference range (6). Given the limitations of retrospective cross-sectional studies with potential for bias associated with choice of anti-androgen treatment, and the fact that T was measured by immunoassay which lacks precision at low serum T concentrations (as opposed to more sensitive liquid chromatography-tandem mass spectrometry (LC-MS), a prospective randomised double-blind trial to evaluate the optimal anti-androgen agent is warranted.

3.3. BACKGROUND INFORMATION

Goals of feminising cross-sex hormone therapy with E2

Male-to-female individuals who opt for gender transition are treated with E2, to achieve E2 levels in a female reference range and lower T levels from a male (10–30 nmol/L) to a female range (< 2 nmol/L). Over 3–24 months, individuals develop softening of skin, a decrease in facial and body hair growth, changes in body fat and muscle distribution (such as a lower waist-to-hip ratio and increased total body fat predominantly in a leg and gynoid region), as

well as breast development. (7) E2 negatively feedbacks to suppress gonadotropin-releasing hormone (GnRH), in turn lowering T to a degree, however most individuals will require treatment with an additional anti-androgen drug (cyproterone acetate or spironolactone).

Anti-androgen therapy

Cyproterone acetate is a steroidal anti-androgen and works in two ways. First, it is a potent peripheral androgen receptor competitive antagonist (IC50 = 7.1 nmol/L) acting at peripheral sites (i.e. skin, hair, body fat, muscle) to decrease the effect of T. Second, it activates the progesterone receptor which, similar to E2 treatment, causes further negative feedback to suppress GnRH and gonadotropins at the hypothalamus/pituitary respectively to lower T production (typically, if added to E2 to < 1 nmol/L) (8, 9). Notably, although used in Europe for many years, cyproterone acetate is not available in the USA for commercial reasons. This confers a distinctive opportunity to study its effects in Australia.

Spironolactone is a mineralocorticoid receptor antagonist and is anti-androgenic in four ways. First, it is a peripheral androgen receptor partial antagonist (IC50 = 77 nmol/L). The IC50, which is the concentration of an inhibitor where the binding is reduced by half, is higher than that of cyproterone acetate, reflecting less potency. Second, Spironolactone is a weak inhibitor of 17α-hydroxylase and 17,20-lyase (enzymes in the T biosynthetic pathway) which lowers T to a small degree. Third, it has weak progestogenic activity with a very low half-maximal potency (10); and fourth, it has estrogenic activity expected to suppress GnRH and gonadotropins at the hypothalamus/pituitary (10). However, even at high doses, small pharmacokinetic studies in healthy males show no significant reduction in serum T (despite a transient rise in FSH and LH in the first 2 days of administration) (11, 12). Interestingly, administration of the main metabolite of spironolactone, canrenone, appears to decrease T by 50–60% within hours in healthy men(13) and in conjunction with E2, spironolactone decreases T (14). As spironolactone may potentially cause feminisation by direct antagonism of the androgen receptor without reduction of serum T, it is unclear which anti- androgen is more effective at feminisation, and prospective studies are required. Whilst both anti- androgen agents, cyproterone acetate and spironolactone, have been shown to lower T levels if added to E2 treatment, there have been no head-to-head studies comparing the superiority of one anti-androgen to another (15).

Breast effects of anti-androgen therapy

Breasts are extremely important for femininity, identity, and self-confidence and an important component of transitioning from male to female. There are no studies comparing cyproterone acetate or spironolactone on breast development. A European observational study demonstrated that only modest increases in breast circumference occur after 1 year of

feminising hormone therapy (16). Noting that relatively low doses of E2 were used (75% below current recommended E2 target range), only 9% developed a bra cup size of A or greater, well below the average female cup size of C. Most breast development occurred in the first 6 months. (16) Although anecdotally, there have been reports that progesterone may enhance breast growth, there are no well-designed studies to assess the effectiveness or refute the lack of effectiveness of progesterone on breast development. (15) As a primary endpoint, we will evaluate the effect of cyproterone acetate, which has progestogenic as well as anti-androgenic activity, on breast size.

Sexual dimorphism and immunity

Sexual dimorphism in immunity refers to the differences that occur in disease incidence and clinical manifestations between males and females. (17) Indeed, studies have shown that males are more susceptible to bacterial, viral, fungal and parasitic infections as well as malignancy than women. (17) Autoimmune diseases also frequently display significant sexual dimorphism, with conditions such as Graves' disease and rheumatoid arthritis more common in women, and others such as ankylosing spondylitis more common in men. (18) This variation has previously been partly attributed to differences in sex chromosomes, sex hormones (such as testosterone, estradiol and progesterone) and epigenetics. (17, 19) Due to the binary definition of sexual dimorphism, there is little understanding of how transgender individuals receiving hormone therapy fit into the current understanding of sexual dimorphism. In particular, the effects of hormone therapy in transgender individuals on innate immune function, susceptibility to infection, malignancy and autoimmune disease are unclear and warrant further investigation. This will be explored in collaboration with colleagues at the Murdoch Children's Research Institute.

4. STUDY OBJECTIVES

4.1. HYPOTHESIS

In male-to-female individuals commencing estradiol therapy for feminisation, additional cyproterone acetate will be more effective than spironolactone in (a) increasing breast volume and feminine fat distribution (increasing gynoid and leg regional fat), and (b) lowering endogenous testosterone levels,

4.2. STUDY AIMS

1. To compare the effectiveness of cyproterone acetate versus spironolactone as an anti-androgen agent used in cross-sex hormone treatments in male-to-female transgender individuals.

4.3. OUTCOME MEASURES

1. Primary Endpoint

- Breast volume

2. Secondary endpoints

- Percentage of individuals who have total T levels < 2 nmol/L (above the female reference range) at 3 and 6 months. ;
- Fat mass including leg and gynoid regional fat;
- Patient Health Questionnaire 9 (PHQ-9) (20)
- Gender dysphoria using the Gender Preoccupation and Stability Questionnaire (GPSQ) (21).

Covariates: Age and Body Mass Index (BMI, kg/m^2)

5. STUDY DESIGN

5.1. STUDY TYPE & DESIGN & SCHEDULE

This is a double-blind, randomised trial in adults aged 16-80 years to compare the anti-androgen effects of 6 months of cyproterone acetate versus spironolactone in 64 male-to-female transgender individuals on E2 therapy. This is a single site study.

<u>STUDY VISITS</u>

Assessment/ Procedure	Screening	Visit 1 (0 months)	Visit 2 (1 month)	Visit 3 (2 months)	Visit 4 (3 months)	Visit 5 (6 months)
Informed Consent	x					
Demographic Information & assessment for eligibility	x					
Clinical assessment including chest circumference		x			x	x
Depression & gender dysphoria questionnaires		x			x	x

**Blood Collection**		x	x	x	x	x
**DXA body composition**		x				x
**Indirect breast anthropometry/ chest photography**		x				x
**Blood pressure (safety measure)**		x			x	X
**Electrocardiograph (ECG)**		x				x

5.2. Standard Care and Additional to Standard Care Procedures

Standard Care Procedures		
Procedure	Time(months)	Dosage/Volume
Anti-androgen drug	0 - 6	As per standard doses
Blood collection	0,1,2,3,6	
DXA	0	0.004 mSv

Additional To Standard Care		
Procedure	Time (months)	Dosage/Volume
DXA	6	0.004 mSv
Questionnaires: PHQ-9 & GPSQ	0,3,6	
Indirect breast anthropometry/ chest photography	0, 6	
ECG	0, 6	

5.3. Randomisation

Participants will be individually randomised by a third party to intervention group 1 (cyproterone acetate 12.5mg daily) or intervention group 2 (spironolactone 100mg daily) in a 1:1 ratio according to a computer generated randomisation procedure, stratified in blocks for baseline age (< 27 and > 27 year-old) and BMI (<24.7 > 24.7 kg/m^2), with cut-offs corresponding to medians found in our previous studies of these individuals. A block size of 2 is chosen, with intervention group allocation randomly permuted and balanced within blocks. Low dose cyproterone 12.5mg has been chosen as this is effective, and reports of meningioma have occurred with higher doses >25mg daily (22). Austin Health Pharmacy Clinical Trials Team will prepare spironolactone 100mg or cyproterone acetate 12.5mg in matching identical capsules. All participants will receive standard care, which will include E2 valerate 4mg daily or equivalent transdermal E2 as an initial dose as per current guidelines (23). A nocte dose will be used so that early morning fasting blood tests will reflect peak serum E2 levels. As per standard clinical care, the E2 valerate dose will be monitored monthly in the first 3 months to ensure serum E2 levels remain in the target range of 250–600 pmol/L (23). The dose will be increased by 2mg each month if levels are below the target range.

5.4. Study methodology

5.4.1. Measurements (performed at baseline, 3 and 6 months)

a. Breast size

Breast development will be assessed by measuring chest and breast circumference to allow calculation of estimated cup size and to allow comparison with other studies . (16) Chest photography will be used to calculate estimated breast volume using the BreastIdea Volume Estimator application using 2D images (24), and 3D imaging using a Microsoft Kinect camera and freely available KScan3D software (version 1.2.02, LMI Technologies, Canada). Photography images will include the area from the top of the shoulders to the bottom of the breast and will not include the face nor any identifying information. Photography images will only be labelled with participant study ID, UR number and date. Patient satisfaction will also be measured using a 5 point Likert-type Scale.

b. Serum Total Testosterone by LC-MS

T levels in the vast majority of studies cited here were measured by immunoassay, which are notoriously inaccurate for quantifying low levels of circulating T (i.e. female range). Comprehensive studies of all available commercial T immunoassays show major method-dependent discrepancies between immunoassays which all deviate from mass-spectrometry (MS)-based methods (25). Newer LC-MS equipment has overcome the previous limitations of

MS technology by providing greatly enhanced sensitivity while retaining reference level specificity. (26) Therefore, LC-MS via a validated NATA accredited laboratory will be used to ensure accurate and robust T results for the study. Serum T will be measured early morning in the fasting state. For E2 valerate titration, serum E2 will be determined using a clinical electrochemiluminescence immunoassay assay (Cobas C8000, Roche Diagnostics). E2 assay has an inter-assay variation of 3.5% at 330 pmol/L and 1.9% at 1800 pmol/L and will be measured at each time point to allow for adjustment of E2 valerate dose as per clinical guidelines.

c. Immune-profiling, Epigenetics & Sexual dimorphism

We will refer deidentified blood samples to the Trained Immunity Epigenetics Team at the Murdoch Children's Research Institute (MCRI) for immune-phenotyping, and epigenetic and transcriptomic profiling of specific innate (monocytes) and adaptive (naïve T and B) cell types. This will evaluate the changes in immune function during sex hormone administration. This is important given that risk of infection is different in genetic males and genetic females, but it is unclear whether this is related to sex hormone concentrations or underlying genetics. Specifically, a participant's leukocytes will be isolated from the buffy coat of a 6mL blood sample and sorted using multi-colour flow cytometry to measure circulating blood cell populations, and cells will be sorted for epigenomic analysis, including: genome-wide DNA methylation (EPIC array), RNA expression (RNA-seq) and histone modification (ChIP-seq). Results of these analyses have no implications for the participant or their family's health or wellbeing.

d. Body Composition by DXA

Body composition to determine regional fat distribution (percentage android, leg and gynoid fat mass, and total fat mass) will be determined by DXA (Prodigy Version 7.51 GE Lunar, Madison, WI) (27).

5.4.2 Safety assessments and withdrawal (performed at baseline, 1,2,3, and 6 months)

Questionnaires to monitor for depression (PHQ-9) and gender dysphoria (GPSQ)(21) will be used. Blood pressure, medical conditions and medication use will be recorded each visit. Serum urea, electrolytes and creatinine will be monitored to assess for risk of hyperkalaemia, dehydration or renal impairment which may occur with spironolactone. An ECG will be performed at baseline and 6 months to monitor the QTc, prolongation of which has been associated with androgen deprivation therapy in men with prostate cancer. (28) Adverse events will be actioned as per Ethics Committee guidelines. Participants will be withdrawn if they meet the criteria for exclusion, or have a change in safety assessments that necessitate

clinical intervention. A further blood test at months 1 and 2 will be obtained to monitor the E2 valerate dose to ensure serum E2 remain in the target range.

6. STUDY POPULATION

6.1. RECRUITMENT PROCEDURE

Given the lack of research, the transgender community are a highly enthusiastic cohort, welcoming of well-designed research studies as we observed during 2017. For this study, we will recruit transgender individuals attending our endocrine clinics. Together with Equinox Gender Clinic (a GP clinic specialising in transgender health), as of December 2016, we have an established database of over 540 individuals regularly attending our clinics. Numbers of new presentations are rapidly increasing each year. Furthermore, close collaborations with the largest GP clinics that specialise in transgender health, namely Equinox Clinic, Northside Clinic and Prahran Market Clinic, as well as the Royal Children's Hospital in Melbourne, have enabled us to recruit 48 individuals newly commencing cross-sex hormone therapy in the last 8 months (6 per month). Based on the trajectory of new presentations attending our clinic, we expect this number to double over the next 2 years. Through active community engagement, we have also recruited 964 transgender adults across Australia to participate in an online transgender health survey.

6.2. INCLUSION CRITERIA

1. Male-to-female individuals diagnosed with gender dysphoria according to the DSM-V criteria who are about to commence treatment with anti-androgen therapy, are on E2 and their baseline total testosterone > 10nmol/L (in the male reference range).
2. Aged from 16 – 80 years (individuals with gender dysphoria choosing to access CHT can do so after the age of 16 without requiring parental consent).

6.3. EXCLUSION CRITERIA

1. Evidence of androgen deficiency at baseline (those below the normal male reference range of T<10nmol/l at our hospital laboratory);
2. Contraindications to spironolactone, cyproterone acetate or E2 therapy including active renal, cardiac, or liver disease, severe depression, hyperkalaemia, thromboembolic disease or estrogen-sensitive malignancy, meningioma, or planned removal of testes during the study;

6.4. CONSENT

Individual informed consent will be obtained from each participant prior to any part of the study being undertaken. Participants will be provided with a copy of the signed Participant Information Consent Form.

7. PARTICIPANT SAFETY AND WITHDRAWAL

7.1. Risk Management and Safety

While we do not anticipate a compromise in safety, there is the risk that tests performed may be distressing (physically or psychologically) or there may be risks that researchers could not reasonably anticipate. Trained staff will perform all tests to minimise risk.

1. **Possible risks associated with taking anti-androgen agents**

Depression is a potential side-effect of cyproterone, although it is unclear whether this is related to the drug itself or the T lowering effect (15). Clinically, benefits of treatment on depression far outweigh any risk. Rare reports of hepatitis have been reported with high-dose (150 ± 50 mg/day) cyproterone acetate. As a safety precaution, we will monitor for depression and liver function and only use low-dose (12.5mg/day). Spironolactone is a diuretic and can cause hyperkalaemia in individuals with renal impairment, although no measurable effect of clinical significance in otherwise healthy individuals. We will monitor blood pressure, renal function (urea, creatinine), and potassium levels to ensure safety. Hyperkalaemia is rare in the setting of normal renal function (no cases observed in our retrospective study [n=38 on spironolactone]) and should not affect blinding. Adverse events will be actioned as per Ethics Committee guidelines. Participants will be withdrawn if they meet the criteria for exclusion, or have a change in safety assessments that necessitate clinical intervention.

2. **Psychological distress**

While we do not envisage this study will cause any participant psychological distress, questionnaires to monitor for depression (PHQ-9 (20)) and gender dysphoria (GPSQ(21)) will be used. These safety assessments will be performed at baseline, 3 and 6 months. Participants will be encouraged to speak to the study doctors if they experience distress. An appropriate level of care will be triaged by the study doctors. This might include withdrawal from the study, referral for treatment at Austin Health or similar health network.

3. **Risks associated with bloods tests**

Venepuncture can cause some discomfort and bruising.

7.2. Handling of Withdrawals

Participants who have chosen to or are asked to withdraw from the study will be reviewed by a study doctor to ensure they are given information about ongoing clinical care. They will be asked to sign the Withdrawal of Participation form (PICF). It will be made clear to them through the trial and at that time that withdrawal will have no negative impact on their relationship with their treating doctors, or Austin Health. No additional personal or health or samples information will be collected from participants once they have withdrawn.

Consistent with an intention-to-treat analysis, any data that has been collected up to the point of withdrawal (of loss to follow up for other reasons) will be included in data analysis. Similarly, all biological specimens will need to be analyzed so that the intention-to-treat principle is not violated.

7.3. Replacements

The study is adequately powered to determine the primary endpoints accounting for 20% attrition and withdrawn participants will not be replaced.

8. Statistical Methods

8.1. Sample Size Estimation & Justification

Based on the percentage of individuals that suppress T<2nmol/L, for a difference of 40% (estimated from our data, 48% in spironolactone group vs 90% in cyproterone), a sample size of 25 per group is required (power 0.9 and level of significance 0.05). Based on a conservative drop-out rate of 20% (our current observational bone health study in transgender individuals has a dropout rate of 15%), it is estimated that a total of 64 participants will be required for enrolment into the study to be powered, to show a difference in total testosterone concentrations between groups. We do not have any preliminary data on breast size.

8.2. Power Calculations

Power calculations are based on the means and standard deviation determined for the secondary endpoint from our preliminary data.

8.3. Statistical Methods To Be Undertaken

We will use a Generalised linear mixed model with a restricted maximum likelihood function to determine between group differences in the main outcome measures over time, adjusted for randomisation strata. Further analysis will include outcomes as a quantitative measure, using Generalised additive mixed models to examine non-linear (spline) effects of hormone treatments over time, adjusted for randomisation strata. The estimated marginal means across each timepoint plus 95% CI between the groups from baseline to study end will be determined, and p values <0.05 are considered statistically significant. Following an intention-to-treat protocol, the analysis will include all randomised subjects who are enrolled in the trial.

9. Storage of Blood and Tissue Samples

9.1. Details of where samples will be stored, and the type of consent for future use of samples

All routine laboratory studies aside from the sex steroid analyses will be performed at Austin Pathology. The results of these tests will go into the participant's Austin Health medical record and be collected separately in their study file. Blood will be discarded within 14 days.

Blood for sex steroid analysis will be collected and couriered to Monash Pathology for analysis via MS/LC-MS. The results of these tests will go into the participant's Austin Health medical record and be collected separately in their study file. The serum estradiol levels will be used for titration of estradiol therapy. Blood samples will be discarded by Monash Pathology within 14 days of analysis.

In collaboration with MCRI, an aliquot of de-identified blood from trial participants will be transferred to MCRI for epigenetic analysis. Blood samples will be discarded within 14 days of analysis.

With additional consent, participants may have their blood stored for future research and genetic testing. The frozen aliquots will be maintained in a locked Austin Health Endocrine Department Laboratory freezer at -80 degrees Celsius indefinitely. These samples will only be labeled with study number and unit record number. Specimen storage will be the responsibility of the PI.

10. Data Security & Handling

10.1. Details of where records will be kept & How long will they be stored

Hard copy information will be stored in a locked filing cabinet within a locked office located in the University of Melbourne Department of Medicine, Austin Health.

An electronic database file on REDCap will be kept for each participant and used in real time during study visits to record data. This will also serve as a way to back up hard copy data and analyse collected data. This will be stored on a password/protected file on a University of Melbourne Department of Medicine Server.

Data collection may include asking the participant to fill out a paper-based health information sheet and questionnaires, or alternatively using REDCap. Forms may be sent to the patient in advance of their appointment to complete and discuss at the appointment. The participant would not be provided with any electronic equipment and can only complete this online method if they have access to a personal computer or device.

Electronic and paper study files will be stored for a minimum of 15 years after the end of the study. After that time, electronic information may be permanently deleted and paper records will be destroyed in Austin Health Confidential Waste bins.

10.2. Confidentiality and Security

Hard copy information will be stored in a locked filing cabinet with a locked office located in the University of Melbourne Department of Medicine, Austin Health.

An electronic database file will also be kept for each participant as back up and a way to analyse this information. This will be stored on a password/protected file on a University of Melbourne Department of Medicine Server.

The code to re/identify participants will be kept in a separate password protected file on a Department of Medicine Server. Access to the University of Melbourne Department of Medicine server requires individual log in details and is controlled and monitored.

10.3. ANCILLARY DATA

Images from scans will be stored in the participant's Austin Health medical record and in their electronic study file. Chest photography images will only be stored in their electronic study file.

References

1. Meerwijk EL, Sevelius JM. Transgender Population Size in the United States: a Meta-Regression of Population-Based Probability Samples. Am J Public Health. 2017;107(2):e1-e8.
2. Rider GN, McMorris BJ, Gower AL, Coleman E, Eisenberg ME. Health and Care Utilization of Transgender and Gender Nonconforming Youth: A Population-Based Study. Pediatrics. 2018.
3. Zucker KJ. Epidemiology of gender dysphoria and transgender identity. Sex Health. 2017;14(5):404-11.
4. Asscheman H, Giltay EJ, Megens JA, de Ronde WP, van Trotsenburg MA, Gooren LJ. A long-term follow-up study of mortality in transsexuals receiving treatment with cross-sex hormones. Eur J Endocrinol. 2011;164(4):635-42.
5. Angus L, Leemaqz S, Ooi O, Cundill P, Silberstein N, Locke P, et al. Cyproterone acetate or spironolactone in lowering testosterone concentrations for transgender individuals receiving oestradiol therapy. Endocr Connect. 2019;8(7):935-40.
6. Liang JJ ea. Testosterone Levels Achieved by Medically Treated Transgender Women in a United States Endocrinology Clinic Cohort. Endocr Pract. 2018;24(2):135-42.
7. Klaver M, de Blok CJM, Wiepjes CM, Nota NM, Dekker M, de Mutsert R, et al. Changes in regional body fat, lean body mass and body shape in trans persons using cross-sex hormonal therapy: results from a multicenter prospective study. Eur J Endocrinol. 2018;178(2):165-73.
8. Gava G, Cerpolini S, Martelli V, Battista G, Seracchioli R, Meriggiola MC. Cyproterone acetate vs leuprolide acetate in combination with transdermal oestradiol in transwomen: a comparison of safety and effectiveness. Clin Endocrinol (Oxf). 2016;85(2):239-46.
9. Toorians AW, Thomassen MC, Zweegman S, Magdeleyns EJ, Tans G, Gooren LJ, et al. Venous thrombosis and changes of hemostatic variables during cross-sex hormone treatment in transsexual people. J Clin Endocrinol Metab. 2003;88(12):5723-9.
10. Fagart J, Hillisch A, Huyet J, Barfacker L, Fay M, Pleiss U, et al. A new mode of mineralocorticoid receptor antagonism by a potent and selective nonsteroidal molecule. J Biol Chem. 2010;285(39):29932-40.
11. Stripp B, Taylor AA, Bartter FC, Gillette JR, Loriaux DL, Easley R, et al. Effect of spironolactone on sex hormones in man. J Clin Endocrinol Metab. 1975;41(4):777-81.
12. Miyatake A, Noma K, Nakao K, Morimoto Y, Yamamura Y. Increased serum oestrone and oestradiol following spironolactone administration in hypertensive men. Clin Endocrinol (Oxf). 1978;9(6):523-33.

13. HC E. Suppression by the spironolactone metabolite canrenone of plasma testosterone in man. Naunyn Schmiedebergs Arch Pharmacol. 1974;285(4):403-6.
14. Prior JC, Vigna YM, Watson D. Spironolactone with physiological female steroids for presurgical therapy of male-to-female transsexualism. Arch Sex Behav. 1989;18(1):49-57.
15. Tangpricha V, den Heijer M. Oestrogen and anti-androgen therapy for transgender women. Lancet Diabetes Endocrinol. 2017;5(4):291-300.
16. de Blok CJM, Klaver M, Wiepjes CM, Nota NM, Heijboer AC, Fisher AD, et al. Breast Development in Transwomen After 1 Year of Cross-Sex Hormone Therapy: Results of a Prospective Multicenter Study. J Clin Endocrinol Metab. 2018;103(2):532-8.
17. Jaillon S, Berthenet K, Garlanda C. Sexual Dimorphism in Innate Immunity. Clin Rev Allergy Immunol. 2019;56(3):308-21.
18. Rubtsova K, Marrack P, Rubtsov AV. Sexual dimorphism in autoimmunity. J Clin Invest. 2015;125(6):2187-93.
19. Saeed S, Quintin J, Kerstens HH, Rao NA, Aghajanirefah A, Matarese F, et al. Epigenetic programming of monocyte-to-macrophage differentiation and trained innate immunity. Science. 2014;345(6204):1251086.
20. McMillan D, Gilbody S, Richards D. Defining successful treatment outcome in depression using the PHQ-9: a comparison of methods. J Affect Disord. 2010;127(1-3):122-9.
21. Hakeem A ea. Development and validation of a measure for assessing gender dysphoria in adults: The Gender Preoccupation and Stability Questionnaire. Int J Transgenderism. 2016;17(3-4):131-40.
22. Ter Wengel P. Meningiomas in three male-to-female transgender subjects using oestrogens/progestogens and review of the literature. Andrologia. 2016;48(10):1130-7.
23. Hembree WC, Cohen-Kettenis PT, Gooren L, Hannema SE, Meyer WJ, Murad MH, et al. Endocrine Treatment of Gender-Dysphoric/Gender-Incongruent Persons: An Endocrine Society Clinical Practice Guideline. Endocr Pract. 2017;23(12):1437.
24. Mikolajczyk M, Kasielska-Trojan A, Antoszewski B. A New Tool for Breast Anthropometric Measurements: Presentation and Validation for Women and Men. Aesthetic Plast Surg. 2019;43(5):1160-70.
25. Sikaris K, McLachlan RI, Kazlauskas R, de Kretser D, Holden CA, Handelsman DJ. Reproductive hormone reference intervals for healthy fertile young men: evaluation of automated platform assays. J Clin Endocrinol Metab. 2005;90(11):5928-36.
26. Harwood DT, Handelsman DJ. Development and validation of a sensitive liquid chromatography-tandem mass spectrometry assay to simultaneously measure androgens and estrogens in serum without derivatization. Clin Chim Acta. 2009;409(1-2):78-84.
27. Cheung AS, de Rooy C, Hoermann R, Gianatti EJ, Hamilton EJ, Roff G, et al. Correlation of visceral adipose tissue measured by Lunar Prodigy dual X-ray absorptiometry with MRI and CT in older men. Int J Obes (Lond). 2016;40(8):1325-8.
28. Gagliano-Juca T, Travison TG, Kantoff PW, Nguyen PL, Taplin ME, Kibel AS, et al. Androgen Deprivation Therapy Is Associated With Prolongation of QTc Interval in Men With Prostate Cancer. J Endocr Soc. 2018;2(5):485-96.
