# Supplementary material for: Effect of Feminizing Hormone Therapy on QTc Interval: A Secondary Analysis of a Randomized Clinical Trial
Source: JAMA Netw Open. 2024 Mar 28;7(3):e243994. doi: 10.1001/jamanetworkopen.2024.3994 (PMC10979315; doi:10.1001/jamanetworkopen.2024.3994)
Supplement: Supplement 2. — Data Sharing Statement [file jamanetwopen-e243994-s002.pdf]

Data Sharing Statement

Angus. Effect of Feminizing Hormone Therapy on QTc Interval: A Secondary Analysis of a Randomized Clinical Trial. JAMA Netw Open. Published online March 28, 2024.
doi:10.1001/jamanetworkopen.2024.3994

Data
Data available: Yes
Data types: Deidentified participant data
How to access data: On reasonable request to the corresponding author Dr Lachlan Angus (lachlan.angus@outlook.com)
When available: With publication

Supporting Documents
Document types: None

Additional Information
Who can access the data: Researchers whose proposed use has been approved
Types of analyses: for a specified purpose
Mechanisms of data availability: after approval of a proposal with signed data access agreement
